# Teflon-on-Glass Molding Enables High-Throughput Fabrication of Hydrophilic-in-Hydrophobic Microwells for Bead-Based Digital Bioassays

**DOI:** 10.3390/ma11112154

**Published:** 2018-11-01

**Authors:** Lisa Tripodi, Karen Ven, Dries Kil, Iene Rutten, Robert Puers, Jeroen Lammertyn

**Affiliations:** 1 Department of Biosystems, Biosensors Group, KU Leuven, 3001 Leuven, Belgium; lisa.tripodi@kuleuven.be (L.T.); karen.ven@kuleuven.be (K.V.); iene.rutten@kuleuven.be (I.R.); 2 ESAT-MICAS, KU Leuven, 3001 Leuven, Belgium; dries.kil@kuleuven.be (D.K.); robert.puers@kuleuven.be (R.P.)

**Keywords:** microfabrication, molding, hydrophilic-in-hydrophobic microwells, Teflon, digital bioassay

## Abstract

In recent years, Teflon-on-glass microwells have been successfully implemented in bead-based digital bioassays for the sensitive detection of single target molecules. Their hydrophilic-in-hydrophobic (HIH) nature enables the isolation and analysis of individual beads, carrying the target molecules, which can be further manipulated accurately through optical tweezer (OT) setups. However, these Teflon HIH-microwell platforms are conventionally fabricated through a complex, time-consuming and labor-intensive dry lift-off procedure which involves a series of major steps, limiting the up-scaling potential of these platforms. Alternative Teflon-based microwell fabrication methods have been extensively explored in literature but they preclude the generation of hydrophobic wells with hydrophilic bottom, thereby hampering the bioassay performance. Here, we present a new Teflon-on-glass molding method for the high throughput fabrication of hydrophilic-in-hydrophobic (HIH) microwell arrays, able to empower bead-based digital bioassays. Microwells 2.95 μm in depth and 3.86 μm in diameter were obtained to host individual beads. In these microwell arrays, sealing of reagents was demonstrated with an efficiency of 100% and seeding of superparamagnetic beads was achieved with an efficiency of 99.6%. The proposed method requires half as many steps when compared to the traditional dry lift-off process, is freely scalable and has the potential to be implemented in different bead-based bioassay applications.

## 1. Introduction

Polytetrafluoroethylene (PTFE), commonly known under the brand name Teflon-AF^®^, is an amorphous fluoroplastics which possesses excellent properties including high optical clarity, low surface energy, high thermal stability and superior chemical and biological inertness. Therefore, it is applied in a variety of devices and fields, ranging from kitchen tools to lab-on-a-chip systems. In particular, Teflon is considered a gold standard in the fabrication of microwell arrays for bead-based digital bioassays, which are biosensing platforms with single-molecule resolution [1,2,3].

In such a bead-based digital bioassay, single target molecules are captured on magnetic beads, which are subsequently compartmentalized in femtoliter-sized microwells. Labeling of the target molecules with an enzyme and co-encapsulation of fluorogenic substrate in the microwells enables the generation of fluorescent product in the confined volume of the microwell. This signal, which indicates the presence of a target molecule on the bead, can then be detected using fluorescence microscopy or other optical detection strategies. As such, the target molecules can be digitally counted.

Multiple microwell-based platforms have been extensively represented in the state-of-the-art for bead-based digital bioassays. Most commonly, microwell arrays have been obtained through chemical polishing of fiber-glass bundles [4], or through fast and simple hot embossing [5,6,7,8,9,10,11], molding [12,13,14] and injection molding [15] of polymers. However, these methods are either not freely scalable for mass production or do not enable proper sealing of the fluorogenic substrate and seeding of the beads (seeding efficiency of ~40%) [15,16,17,18], both key factors for bead-based digital bioassay performance and reliability.

To improve these key concepts, alternative microwell arrays were fabricated with a hydrophobic upper surface, to allow droplet movement over the chip, and hydrophilic bottom surfaces, enabling efficient confinement of both aqueous solutions and beads. Although initial studies on these hydrophilic-in-hydrophobic (HIH) microwells [12,19,20] reported improved reagent sealing and bead seeding efficiencies (50–60%), the real breakthrough in terms of bioassay performance came with the introduction of HIH Teflon-based microwells arrays [21], ensuring complete reagent sealing and seeding efficiencies up to 99%. As such, these HIH Teflon microwells have been shown to successfully enable the detection of single target molecules, including DNA, proteins, and even cells [22,23,24,25]. Moreover, we recently demonstrated the successful implementation of these HIH microwells with digital microfluidics and an optical tweezer (OT) setup for the accurate manipulation of individual magnetic beads, carrying a target molecule of interest for further analysis [21,26]. Consequently, HIH Teflon-based microwells have proven to be the current gold standard for reliable bead-based digital bioassays and to be widely applicable, ranging from biosensing to life-science applications.

However, Teflon-based HIH-microwells are fabricated through a dry lift-off procedure [27], involving a series of 6 major steps including chemical vapor deposition of Parylene-C, thermal evaporation of aluminum, photolithography, wet etching of aluminum, reactive ion-etching and peel-off. As such, this is a complex, time-consuming and labor-intensive procedure with limited up-scaling potential. In this context, several methods have been recently reported in an attempt to provide a scalable fabrication technique for HIH microwells. These techniques rely on single-step imprinting [28] or soft-lithography of hydrophobic polymers on hydrophilic substrates [29]. Although this enables fast fabrication of HIH microwells, the resulting wells are not equals of the Teflon-based HIH microwells, as (i) the presence of squeeze film of the hydrophobic polymer at the bottom of the well cannot be completely excluded, hence potentially jeopardizing the HIH nature of the wells or (ii) these polymers have not been shown to be match Teflon in terms of surface inertness, thermal stability and optical clarity.

In this work, we combine the concept of Teflon-on-glass surfaces and an embossing-like procedure into a fast and simple approach for fabricating HIH microwells, embracing the advantages of conventional HIH Teflon-based microwells with the scalability of hot embossing and molding methods. The process relies on the molding of a spin-coated Teflon layer on a glass substrate with a reusable silicon micropillar mold using a Flip Chip machine, commonly used for wafer to wafer bonding. First, we describe the proposed fabrication process and characterize the geometrical features of the resulting microwells. Next, we evaluate the characteristics of the resulting Teflon surface through contact angle measurements and atomic-force microscopy (AFM), and further optimize the fabrication procedure. Finally, we show successful sealing of aqueous reagents and seeding of magnetic beads, demonstrating the potential of these microwells for the implementation of bead-based digital bioassays.

## 2. Materials and Methods

### 2.1. Silicon Micropillar Mold Fabrication and Characterization

**Figure 1:**
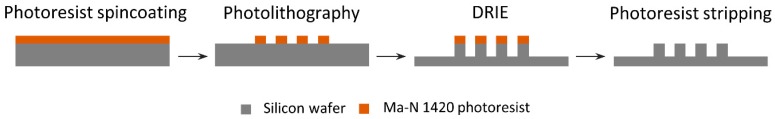
Schematic representation of the proposed micropillar mold fabrication procedure, including photoresist spin-coating, photolithography, DRIE and stripping of the photoresist.

The proposed procedure for fabricating silicon micropillar molds is illustrated in Figure 1. Silicon wafers (3 inch, 100 ± 5°, p-type, Si-Mat Silica Materials) were first submersed for 5 min in 2% hydrofluoric acid (HF) to remove native silicon dioxide. Next, the wafers were rinsed with deionized water, dried with a nitrogen gun and baked for 2 min on a hotplate at 250 °C to remove residual water. Subsequently, Ma-N 1420 negative photoresist (Microresist Technology, Berlin, Germany) was spin-coated on the wafers at 1500 rpm for 30 s (Spin-coater Headway Research, Inc., Daejeon, Korea) and baked at 100 °C for 2 min (final photoresist thickness of 2 µm). A chromium-glass mask (DeltaMask, Enschede, the Netherlands), bearing 3 arrays of 10,000 micro-holes each (3 µm diameter), was applied when exposing the photoresist to UV light (EVG620 illuminator, 365 nm) at a dose of 850 mJ/cm^2^. Next, the photoresist was developed in ma-D 533/S (Microresist Technology, Berlin, Germany) for 90 s, and the wafers were rinsed with deionized water and dried with a nitrogen gun. The exposed silicon surface was subsequently etched by deep reactive ion etching (DRIE, OXFORD Instruments, Abingdon, UK) through a Bosch procedure. Finally, the wafers were cleaned through sonication in acetone to strip photoresist residuals. 2D and 3D characterization of the silicon micropillar mold was performed using a 3D optical microscope (Sensofar Metrology, Barcelona, Spain).

### 2.2. Microwell Array Fabrication and Characterization

First, borofloat glass wafers (3-inch glass wafers, SCHOTT, Mainz, Germany) were washed for 2 min in acetone, isopropanol and deionized water and dried with a nitrogen gun. Next, the wafers were subjected to a short plasma cleaning, followed by fluoroalkylsilane (Dynasylan F8263, Evonik, Germany) spin-coating at 3000 rpm for 15 s. Subsequently, the wafers were rinsed with isopropanol, dried with a nitrogen gun and baked for 10 min on a hotplate at 110 °C. Teflon-AF^®^ (Chemours, Wilmington, DE, USA) 6% w/w was spin-coated on the glass wafers at 500 rpm for 60 s.

**Figure 2:**
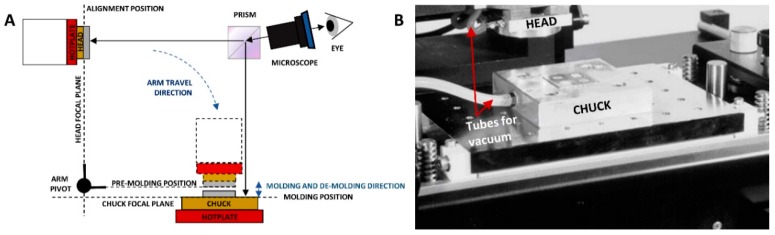
(**A**) Schematic representation of the working principle of the FCM device, used for molding. After alignment of the samples, both fixed to the FCM head and chuck using vacuum, the head is rotated from the alignment position to the pre-molding position, after which it is moved downward into the molding position for molding. After molding, the head is moved back up into the pre-molding position and subsequently rotated into the alignment position. (**B**) Picture of the FCM device, including the head, chuck and tubes for vacuum. Adapted with permission from [30].

A Flip Chip machine (FCM tool, Karl Suss MicroTec, Garching, Germany), depicted in Figure 2, was used to mold the Teflon-coated glass wafers with the silicon micropillar molds with high pressure and high alignment accuracy. First, the FCM head and chuck were preheated to 110 °C using the connected hotplates (Figure 2A). Next, the silicon micropillar mold was loaded on the FCM head and the Teflon-coated glass wafer was loaded on the FCM chuck. Vacuum was applied to the head and chuck to properly fix the silicon micropillar mold and wafer (Figure 2B). As such, the Teflon-coated glass wafer was baked at 110 °C for 1 min.

Meanwhile, the silicon mold and the Teflon-coated glass wafer were manually aligned using the microscope with a split prism and the silicon micropillar mold was positioned above the wafer by rotating the FCM arm (equipped with a pivot) from the alignment position to the pre-molding position (Figure 2A). After completing the 5 min baking step at 110 °C, molding was performed by moving the FCM head down in a perpendicular direction, from the pre-molding to the molding position to physically contact the wafer. Next, force was slowly increased from 0 to 138 N, which was then maintained for 5 min at a temperature of 110 °C. De-molding of the silicon mold was performed by decreasing the force from 138 to 0 N, while keeping the temperature at 110 °C. Afterwards, the FCM head was lifted perpendicularly to the pre-molding position and the FCM arm was moved back to the alignment position. Finally, the vacuum of the chuck was switched off to unload the molded wafer.

**Figure 3:**
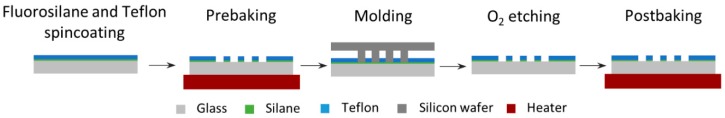
Schematic representation of the microwell array fabrication procedure, including fluoroalkylsilane and Teflon spin-coating, prebaking, molding, O_2_ etching and post-baking.

Following molding, samples were either used as such (Procedure I) or subjected to a variety of additional post-treatment steps, resulting in 7 potential fabrication procedures in total, as listed in Table 1. In Procedures II to VII, an additional O_2_ reactive ion etching (RIE) step (100 Watt, 40 sccm, 50 mTorr, 50 s) was performed after molding. Afterwards, samples of Procedures III to VII were post-baked at different temperatures above the glass transition temperature for different times. The post-baking step of procedure VII was performed by flipping the sample upside down. Additionally, a reference Teflon chip (REF) was prepared, which was baked according to the manufacturer’s instructions [31] and not subjected to molding or plasma treatment. A schematic representation of the fabrication steps, used in the different procedures, is shown in Figure 3.

The morphology of the resulting microwells was characterized through 2D profile-sections and 3D images, obtained with a 3D optical microscope (Sensofar Metrology, Barcelona, Spain). The average and standard deviation of 3 randomly selected wells was calculated. The surface wettability of the samples was characterized by measuring the static contact angle of 4 droplets of 9 µL Milli-Q water (Sartorius Stedim, Biotech SA, Goettingen, Germany), randomly placed on the Teflon surface using a Pendant Drop device (KSV Instruments, Helsinki, Finland) and analyzed with a Laplace-Young curve fitting (CAM 2008 software, KSV Instruments, Helsinki, Finland). Topographical characterization of the samples surfaces was performed using an atomic force microscope (Pico LE 1-AFM, Agilent AFM System, Newton, NJ, USA). The scanned area was 25 µm^2^ and the resulting images were analyzed using a SPIP^TM^—Scanning Probe Image Processor Software (Image Metrology, version 6.7.4).

### 2.3. Bead Seeding and Reagent Sealing

Sealing of reagents in the HIH microwells was evaluated by applying a 30 µL droplet of fluorescein (150 µM) dissolved in DI water on top of the array, which was then covered with a 150 μL droplet of Plus One Drystrip Coverfluid oil (GE Healthcare, Europe GmbH, Diegem, Belgium). Subsequently, the droplet of fluorescein was carefully dragged away from underneath the oil with a pipet tip, resulting in femtoliter-sized reaction wells, filled with fluorescein and covered with oil. These fluorescent droplets were visualized using an inverted fluorescence microscope (IX71, Olympus Corporation, Tokyo, Japan) and EM-CCD camera (Hamamatsu Photonics K.K., Shizuoka, Japan). Imaging was performed using a 40× objective, WIBA filter (excitation BP460-495, emission BA510-550), dichromatic filter (DM505) and neutral density filter (3%). These tests were performed on samples, fabricated through Procedure I but without the addition of a layer of fluoroalkylsilane, through Procedure VII and through the conventional dry lift-off procedure [21].

Superparamagnetic beads (Lodestar 2.7 Streptavidin, Agilent Technologies Belgium, Diegem, Belgium) were seeded in the HIH microwells following a previously described procedure [23,24].

In short, a 5 μL droplet of Superblock buffer containing superparamagnetic beads was manually shuttled back and forth 20 times over the microwell array using a pipet tip, while positioning the array on top of a magnet (NdFeB, 16 mm^2^ area, 12.7 N, Supermagnete, Gottmadingen, Germany). Next, the array was visualized using bright field microscopy (Nikon TiEclipse, Tokyo, Japan) with a 40× magnification objective. These tests were performed on samples fabricated through Procedure VII.

Sealing and seeding efficiencies (i.e., the percentage of wells sealed, and beads seeded respectively), were calculated by normalizing respectively the number of fluorescent wells or seeded beads to the total number of wells. These values were obtained by analyzing the microscopy images using ImageJ software (US National Institutes of Health, Bethesda, MD, USA).

## 3. Results and Discussion

### 3.1. Silicon Micropillar Mold Fabrication and Characterization

Micropillar molds were fabricated using the previously described DRIE-Bosch procedure. Different molds with pillars of a particular height were fabricated to evaluate the influence of the space in between the Teflon surface and the base of the mold, required for solvent evaporation from the Teflon solution while molding. To obtain the different pillar heights between molds, the wafers were treated with different iterations of the Bosch passivation/etch cycles (i.e., 25, 50, 100, 200 and 250 cycles). This resulted in molds with pillars of 3.50 ± 0.15 µm, 6.67 ± 0.03 µm, 15.56 ± 1.15 µm, 22.66 ± 1.52 µm and 31.00 ± 1.72 µm after photoresist removal.

**Figure 4:**
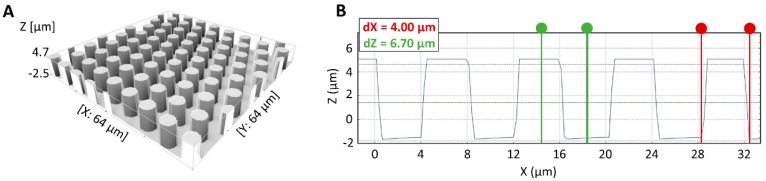
(**A**) 3D image of a silicon micropillar mold. (**B**) 2D section of silicon micropillar mold with a pillar of 4.00 µm in diameter at their bottom (indicated in red) and 6.70 µm on average height (indicated in green) after the sonication process in acetone.

An example of a silicon micropillar mold, obtained after 50 iterations, is represented in Figure 4. The mold contains 3 arrays, each of them comprising 10,000 pillars (slightly conical) with an average height of 6.67 ± 0.03 µm, an average diameter of 4.01 ± 0.12 µm at the bottom, 3.80 ± 0.20 µm in the center and 3.50 ± 0.10 µm at the top.

### 3.2. Microwell Array Fabrication and Characterization

Molding of the microwells was performed using an FCM device, as described above. The split prism in the FCM device enabled proper alignment of the mold and substrate to (1) ensure equal force distribution among the silicon micropillars during molding, (2) reflect the shape of their sidewalls to the Teflon wells, and (3) ensure molding reproducibility.

**Figure 5:**
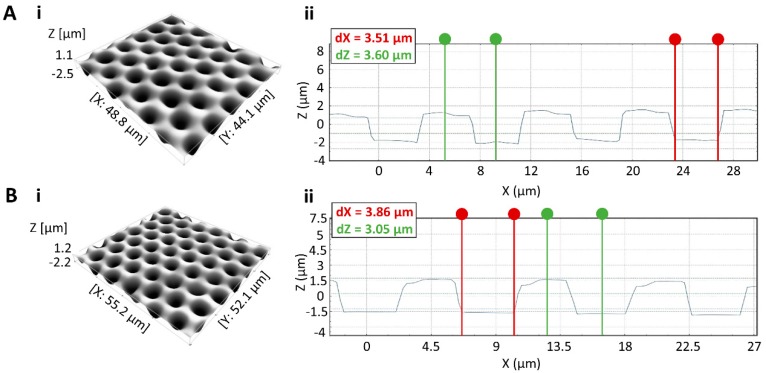
(**A**) Microwell array fabricated by molding (Procedure I). (i) 3D image of the microwell array, and (ii) 2D section of the molded microwell array with a well of 3.51 μm in diameter (indicated in red) and another well of 3.60 µm in depth (indicated in green). (**B**) Microwell array, fabricated by molding and subsequent plasma treatment (Procedure II). (i) 3D image of the microwell array, and (ii) 2D section of the microwell array after plasma treatment, resulting in removal of Teflon squeeze film from the bottom of the wells and minimally affecting the well geometry, with a diameter of 3.86 µm (indicated in red) and depth of 3.05 µm (indicated in green).

First, the performance of the molds with different pillar heights was evaluated. Teflon layers were spin-coated on glass slides at 500 rpm for 60 s, resulting in a layer thickness of 2.9 μm ± 0.18 μm. The shortest pillars (3.5 μm) were found to hamper complete solvent evaporation during molding, as the Teflon was not completely solidified after the procedure with the polymer surface remaining partially liquid and sticky. Contrary to the lack of room for solvent evaporation with the smallest pillars, partial or complete pillar breakage was observed during molding or de-molding for the pillars of 15.56, 22.66 and 31.0 µm. Therefore, microfabrication was continued with pillars of 6.67 µm, based on the trade-off between pillar strength and room for solvent evaporation during molding. Figure 5A shows a microwell array, obtained after molding (Procedure I, Table 1). The resulting microwells had an average diameter of 3.51 ± 0.16 µm, in compliance with the micropillar mold, and were on average 3.43 ± 0.14 µm deep, when taking into account the presence of a bump of approximately 300 nm at the circumference of each microwell. This elevation is imputable to a portion of the volume of Teflon, moved by each individual pillar during the molding process to create the well. The presence of these bumps, however, did not hinder droplet transport on the chip nor sealing of reagents and seeding of the bead, as will be illustrated in a following section.

While the shape of the wells corresponded to the requirements for implementing bead-based bioassays, the presence of hydrophobic fluoroalkylsilane at the bottom of the microwells was able to hinder the desired hydrophilicity in the well bottom. Moreover, the presence of Teflon squeeze film at the bottom of the wells could not be excluded. To remove these hydrophobic layers and ensure the true HIH characteristics of the microwells, an O_2_ RIE plasma treatment [32] was performed (Procedure II, Table 1). However, this plasma treatment was expected to contribute not only to the specific removal of the unwanted hydrophobic layers at the bottom of the wells, but also to the removal of Teflon at the inter-microwell surface and the microwell walls. To restrict the thus-induced distortion of the microwell geometry, an optimal etching time of 50 s was established. As depicted in Figure 5B, this plasma treatment resulted in complete removal of the Teflon squeeze film (illustrated by the flat well bottom) while minimally deforming the microwells (average depth decreases to 3.03 ± 0.03 µm and average diameter increase to 4.03 ± 0.14 µm), hence making it possible to host a single magnetic bead of 2.7 µm in diameter in a single well. Conversely, shorter and longer O_2_ etching times were found unfit for the purpose as they respectively resulted in incomplete removal of the Teflon squeeze film or in excessive enlargement of the microwells, enabling isolation of more than one bead and hence impeding the implementation of a bead-based digital bioassay (data not shown).

### 3.3. Contact Angle and AFM Measurements

Although the decreased well depth and increased diameter does not pose issues for performing digital bioassays, plasma etching of Teflon is known to affect the chemical and structural characteristics of the polymer surface, altering its surface energy and thus hydrophobicity [33]. As this might impair droplet movement, which is crucial for digital bioassays, the wettability and structuring of the resulting Teflon surface has to be evaluated. In particular, the RIE procedure is known to insert structural modifications in the surface by patterning it in a repetitive way on a nm-scale. Reports demonstrate that this structuring could improve the surface hydrophobicity. In contrast, the chemical modifications of the surface, induced by O_2_ plasma etching, are known to have the opposite effect, since by increasing the number of OH groups, the Teflon surface is rendered more hydrophilic [33]. To quantify the effect of the O_2_ plasma etching on the surface wettability, which is crucial for proper bioassay performance, static contact angles were measured on a Teflon reference sample (REF, Table 1) and samples after molding (Procedure I, Table 1) and after O_2_ plasma etching (Procedure II, Table 1).

**Figure 6:**
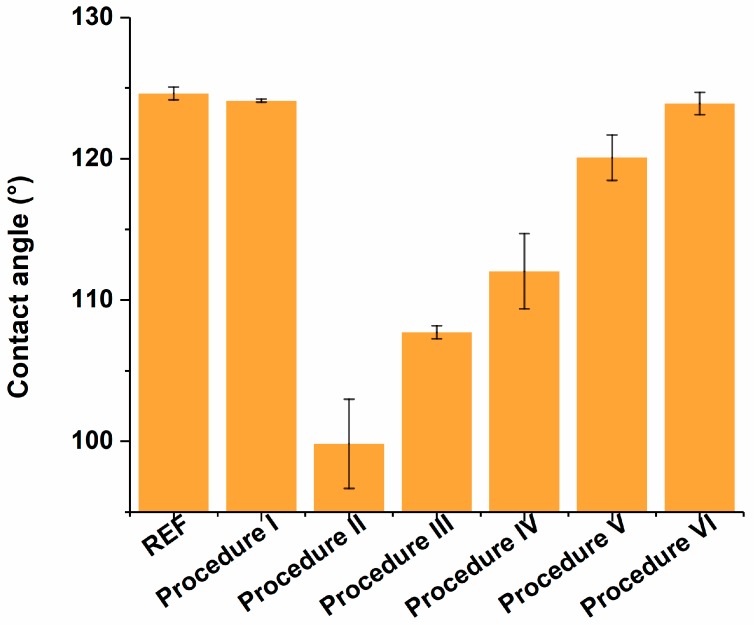
Contact angle measurements of a reference Teflon surface (REF) and the samples fabricated through 6 different procedures (Procedures I to VI). Error bars represent the standard deviation over 4 repetitions.

As depicted in Figure 6, the molding procedure itself, without any post-treatment, did not cause a significant change in the surface energy when compared to the reference surface. However, after plasma treatment, a decrease in contact angle from 124.63 ± 0.45° to 99.83 ± 3.15° was observed, illustrating the decrease in hydrophobicity of the Teflon surface. As the resulting surface is not hydrophobic enough to ensure smooth droplet movement, this impairs the implementation of a bead-based bioassay. Therefore, recovery of the hydrophobicity is desired, which has been previously established by allowing the chips to restore either by ageing with time through the polymers’ surface adaption and reptation [33], or by speeding up polymer-chain reflowing by heating the polymer above its glass transition temperature (T_g_) [34].

As the aim of this research is to establish a simple and fast alternative microwell fabrication procedure, the second approach was pursued as to speed up the Teflon surface recovery step. For this purpose, a variety of post-baking steps, in which the samples were heated above the T_g_ of Teflon (T_g_ = 160 °C) were characterized through contact angle measurements. These procedures include a baking step of 10 min at 165 °C, which is 10 °C above the boiling points of the polymer solvents (Procedure III, Table 1), 10 min at 250 °C, which is 90 °C above the T_g_ of Teflon (Procedure IV, Table 1), for 15 min at 250 °C (Procedure V, Table 1) or for 10 min at 165 °C, followed by 2 min at 330 °C (Procedure VI, Table), as suggested by the manufacturer. As illustrated in Figure 6, most of the thermal procedures resulted in partial surface energy recovery, whereas complete restoring of the surface energy was not achieved. The latter can be due to partial cross-linking of Teflon free C-C radicals during plasma treatment, which may induce surface hardening [35].

**Figure 7:**
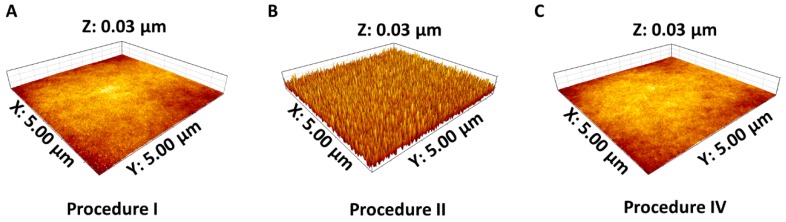
AFM images of samples after (**A**) molding (Procedure I), (**B**) plasma etching (Procedure II), and (**C**) post-baking at 250 °C for 10 min (Procedure IV).

In particular, impaired manual droplet movement was observed on the chips prepared according to Procedure III, thus rendering them useless for bioassay implementation. In addition, the baking steps of Procedures V and VI, which performed significantly better than the others in terms of contact angle, resulted in the complete loss of the microwells, as excessive reflowing of the bulk polymer chains caused refilling of the microwells. Therefore, fabrication Procedure IV was considered most optimal. To confirm that structural alterations, induced by the O_2_ plasma, were restored through the post-baking steps and that the persisting surface energy alterations after post-baking were caused by chemical modifications of the surface only, AFM measurements were performed on the samples, prepared according to Procedures I, II and IV (Figure 7). In terms of roughness, results indicate an average height of 1.16 nm for samples after molding (highest rough point: 2.31 nm, Procedure I), which increased to 13.49 nm after O_2_ plasma etching, with 27.17 nm as the highest rough point (Procedure II). Following the 10-minute post-bake step at 250 °C (Procedure IV), the average roughness height decreased back to 1.30 nm (highest rough point: 2.60 nm), indicating successful restoration of structural modifications and confirming that the persisting surface energy alterations have a chemical origin.

**Figure 8:**
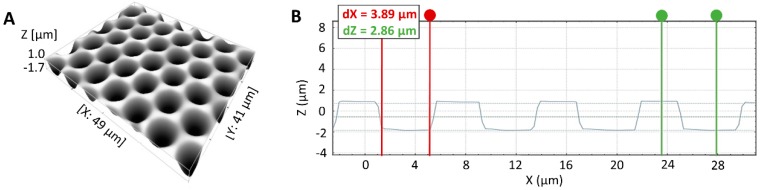
Microwell array, fabricated by molding, plasma etching and baking the sample upside down for 10 min at 250 °C (Procedure VII). (**A**) 3D image of the microwell array. (**B**) 2D section the microwell array with a well of 3.89 µm in diameter (indicated in red) and 2.86 µm in depth (indicated in green).

While the baking step in Procedure IV did not result in complete loss of the microwell array, in-depth 3D visual evaluation of the microwells also revealed deformation of the microstructures. In particular, due to thermal reflowing of the polymer, the overall well depth and diameter was consistently reduced to less than 2.5 µm (data not shown). To overcome this undesired effect, which could impede isolation of single beads in the microwells, the reflowing step at 250 °C was altered by baking the samples upside down, to reduce the gravitation-induced filling of the microwells with the reflowing polymer. As can be seen in Figure 8, this approach prevented microwells from shrinking excessively (average diameter of 3.86 ± 0.06 µm and depth of 2.95 ± 0.08 µm) and resulted in microwells which are tailored to host a single magnetic bead 2.7 µm in diameter, thus showing great potential for implementation in digital bioassays.

### 3.4. Seeding and Sealing Efficiency

**Figure 9:**
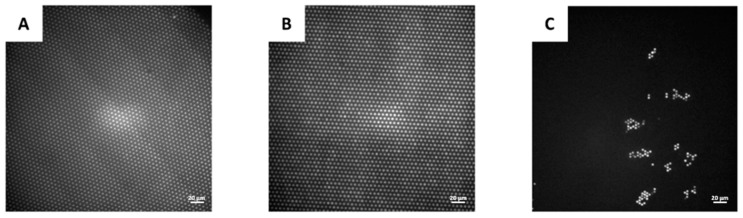
Fluorescence microscopy images of sealing an aqueous fluorescein solution in microwells with 40× magnification. (**A**) Sealing efficiency of nearly 100% in HIH microwells, fabricated through the proposed fabrication procedure (Procedure VII). (**B**) Sealing efficiency of nearly 100% in HIH microwells, fabricated through the conventional dry lift-off procedure. (**C**) Sealing efficiency of 3.79% in microwells, fabricated through molding without plasma etching (Procedure I), in the absence of fluoroalkylsilane.

To further evaluate the HIH properties of the microwells and evaluate their applicability in digital bioassays, their potential to seal aqueous solutions was evaluated. For this purpose, an aqueous solution of fluorescein was applied on top of the HIH microwell array, covered with oil and subsequently removed from underneath. As such, droplets of fluorescein were isolated in the microwells and protected from evaporation by the oil. As illustrated in Figure 9A, a sealing efficiency of 100% was obtained, which is comparable to the sealing in HIH microwells, fabricated through the conventional dry lift-off procedure (Figure 9B). Moreover, impaired sealing of the wells due to the presence of Teflon squeeze film in the bottom of the wells after molding is illustrated in Figure 9C. This sample was prepared without fluoroalkylsilane, to preclude sealing issues because of the presence of the hydrophobic silane at the well bottom.

**Figure 10:**
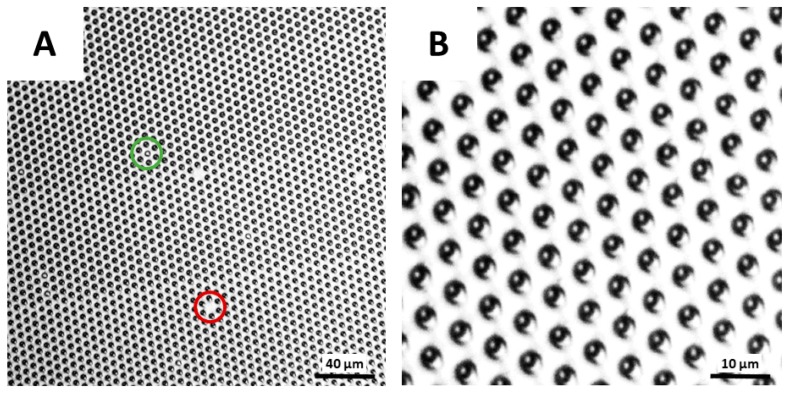
Bright-field microscopy images of seeding superparamagnetic beads in microwells. Seeding efficiency of 99.6% in HIH microwells, fabricated through the proposed fabrication procedure (Procedure VII) with (**A**) 40× and (**B**) 65× magnification. The green circle indicates an empty well (i.e., microwell without bead), while the red circle indicates a defect in the array (i.e., absent microwell).

In addition, the possibility of seeding magnetic beads in the HIH microwells was evaluated. To do so, a magnet was placed below the array, and a 5 µL droplet, containing superparamagnetic beads, was manually shuttled back and forth over the array for two times. Then, bright-field images were recorded, and the seeding efficiency was calculated as the ratio of the number of wells containing one bead to the total number of wells in the field of view. As such, a seeding efficiency of 99.6% was obtained (Figure 10), which is comparable to previously reported seeding efficiencies in the microwells, fabricated using the conventional dry lift-off procedure [21] and drastically larger than seeding efficiencies, obtained through other fabrication techniques [8,9,10,11,12,19,20]. Please note that the defects, indicated with red circles and imputable to artefacts during the lithographic step, should not be confused with empty wells, indicated with green circles, as only the latter contribute to the seeding efficiency calculation. Together, these results demonstrate the successful fabrication of Teflon-based HIH microwells using the newly proposed molding procedure, showing the great potential of these microwells for implementation in digital bioassays.

## 4. Conclusions

In this work, a fast and robust way of fabricating Teflon-based HIH microwells was demonstrated. Microwells of 2.95 μm in depth and 3.86 μm in diameter were obtained through molding with a silicon micropillar mold. Short oxygen plasma treatment enabled removal of hydrophobic fluoroalkylsilane and Teflon squeeze film from the bottom of the microwells. A subsequent upside-down reflowing step at 250 °C made it possible to recover the surface energy, without deteriorating the morphology of the wells. Sealing of aqueous droplets was demonstrated with an efficiency of nearly 100% using fluorescein. Moreover, seeding of superparamagnetic beads was achieved with an efficiency of 99.6%, demonstrating the potential of the HIH microwells for implementation in digital bioassay applications.

As such, the presented work shows a faster, cheaper and robust molding-based alternative to the conventional dry lift-off process for fabricating Teflon-based HIH microwell arrays, as it reduces the number of major processing steps from 6 to 3 (molding, O_2_ plasma etching and post-baking) and the fabrication cost by obviating the need for sophisticated and costly devices. The proposed method only relies on such tools (i.e., DRIE device) for the fabrication of the Si-micropillar mold which, however, can be re-used multiple times to generate a large number of Teflon-on-glass HIH microwells. In addition, the Flip Chip device and plasma etching tool required for the presented fabrication method can be implemented in a non-cleanroom facility, thereby further reducing the production cost.

The presented approach embraces several advantages all at once: (i) it takes advantage of Teflon properties (surface inertness, thermal stability, etc.), (ii) it addresses HIH properties, ensuring reliability and reproducibility (i.e., it makes it possible to ensure a homogeneous hydrophilicity at the well bottom due to the quick plasma procedure thus removing squeeze film), and (iii) it is scalable for mass production, as it relies on a silicon micropillar mold which can be reused multiple times for the continuous generation of Teflon microwells. Moreover, since it relies on direct imprinting using a rigid silicon micropillar mold, by selecting other lithography procedures, the feature size could be further reduced from micrometrical down to nanometrical dimensions, in contrast to the previously reported molding procedures, which rely on elastomer-based molds (e.g., Polydimethylsiloxane/PDMS) [28], thereby limiting the feature dimensions to the micrometer scale.

Finally, the newly fabricated microwells are easily implementable in different microfluidics and OT technologies, and as such, are widely applicable in both biosensing and life-science frameworks.

## Figures and Tables

**Table 1:** List of the steps of different microwell array fabrication procedures, following spin-coating of the fluoroalkylsilane and Teflon on the glass substrate. The procedure steps include molding, plasma treatment and 1 or 2 post-baking steps. Additionally, the fabrication procedure of a reference Teflon sample (REF) is included, prepared according to the manufacturer’s instructions.

Procedure	Molding	Plasma	Post-Bake 1	Post-Bake 2
Procedure I	110 °C	-	-	
Procedure II	110 °C	Yes	-	-
Procedure III	110 °C	Yes	10 min at 165 °C	-
Procedure IV	110 °C	Yes	10 min at 250 °C	-
Procedure V	110 °C	Yes	15 min at 250 °C	-
Procedure VI	110 °C	Yes	10 min at 165 °C	2 min at 330 °C
Procedure VII	110 °C	Yes	10 min at 250 °C ↕	-
REF	-	-	5 min at 110 °C	10 min at 250 °C

The ↕ symbol indicates flipping of the sample.
